# Comparing the effectiveness and cost-effectiveness of text-message reminders and telephone patient navigation to improve the uptake of faecal immunochemical test screening among non-responders in London: a randomised controlled trial protocol

**DOI:** 10.1136/bmjopen-2023-079482

**Published:** 2024-06-22

**Authors:** Thomas Duffy, Natalie Gil, Benzeer Siddique, Stephen Duffy, Andrew Prentice, Sarah Marshall, Natasha K Djedovic, Michael Lewis, Josephine Ruwende, Christian von Wagner, Robert Kerrison

**Affiliations:** 1 University College London, London, UK; 2 Department of Behavioural Science & Health, University College London, London, UK; 3 Health Sciences, University of Surrey Faculty of Health and Medical Sciences, Guildford, UK; 4 NHS England, Redditch, UK; 5 CRUK Department of EMS, Woflson Institute of Preventive Medicine, London, UK; 6 St Mark’s Bowel Cancer Screening Centre, London North West Healthcare NHS Trust, Harrow, UK; 7 St Mark’s Bowel Cancer Screening Centre, London North West Healthcare NHS Trust, London, UK; 8 Northwick Park Hospital, NHS Bowel Cancer Screening Programme London Hub, London, UK; 9 University of Birmingham, Birmingham, UK

**Keywords:** Patient Navigation, Mass Screening, Randomized Controlled Trial

## Abstract

**Introduction:**

Participation in bowel cancer screening is lower in regions where there is high ethnic diversity and/or socioeconomic deprivation. Interventions, such as text message reminders and patient navigation (PN), have the potential to increase participation in these areas. As such, there is interest in the comparative effectiveness of these interventions to increase bowel cancer screening participation, as well as their relative cost-effectiveness.

**Methods and analysis:**

This study will use a three-arm randomised controlled trial design to compare the effectiveness and cost-effectiveness of text message reminders and PN to increase the uptake of bowel cancer screening in London. Participants will be individuals who have not returned a completed faecal immunochemical test kit within 13 weeks of receiving a routine invitation from the London bowel cancer screening hub. Participants will be randomised (in a 1:1:1 ratio) to receive either (1) usual care (ie, ‘no intervention’), (2) a text message reminder at 13 weeks, followed by repeated text message reminders at 15, 17 and 19 weeks (in the event of non-response) or (3) a text message reminder at 13 weeks, followed by PN telephone calls at 15, 17 and 19 weeks in the event of non-response. The primary endpoint will be participation in bowel cancer screening, defined as ‘the return of a completed kit by week 24’. Statistical analysis will use multivariate logistic regression and will incorporate pairwise comparisons of all three groups, adjusted for multiple testing.

**Ethics and dissemination:**

Approvals to conduct the research have been obtained from University College London’s Joint Research Office (Ref: 150666), the Screening Research, Innovation and Development Advisory Committee (‘RIDAC’, Ref: 2223 014 BCSP Kerrison), the Health Research Authority (Ref: 22/WM/0212) and the Confidentiality Advisory Group (Ref: 22/CAG/0140). Results will be conveyed to stakeholders, notably those managing the screening programme and published in peer-reviewed journals/presented at academic conferences.

**Trial registration number:**

ISRCTN17245519

**STRENGTHS AND LIMITATIONS OF THIS STUDY**

- This is a randomised controlled trial using individual-level data, as opposed to area-level data, increasing the studies ability to attribute increases in uptake, directly to the intervention.
- The study focuses on ethnically diverse areas, increasing the generalisability of the research to other parts of the UK.
- The study relies on general practices to consent to the study, which introduces an element of self-selection bias.
- The text message reminders will be delivered in English, which may limit the equitability of the intervention and undermine its effectiveness.
- Not every participant will have a mobile telephone number, which could undermine results. The study will use intention-to-treat analysis to compare individuals across groups, who have a mobile number recorded on the general practitioner system.

## Introduction

 Colorectal cancer (CRC, also known as ‘bowel cancer’) is a leading cause of cancer-related mortality in Europe.^1^ ^2^ Several large randomised controlled trials (RCTs) have shown that biennial faecal immunochemical test (FIT) screening can improve CRC outcomes by detecting cases early.^3^ In light of this, many countries, across Europe, have implemented programmes based on the test.^4^

England is one such country that has implemented an FIT-based programme. In England, the National Health Service (NHS) began offering biennial FIT screening to adults aged 60–74 years in 2019. The screening programme, referred to as the NHS Bowel Cancer Screening Programme (BCSP), is delivered through five national hubs, which send kits to patients registered with a general practice within their respective regions. The delivery of invitations is an automated and centralised process in which information held by the patient’s general practice, including their date of birth, name and address, is shared with the BCSP to facilitate the process. Date of birth is used to identify individuals who are eligible for screening, while name and address are used to send the invitations to those individuals. An age extension to the programme is currently underway, with the aim of all adults aged 50–59 receiving an invitation by 2025. At the time of writing, the programme has been extended to include adults aged 54–59 years.

As with any screening programme, achieving the public health benefits of bowel cancer screening is highly dependent on high and equitable uptake. However, despite being offered free at the point of use, in England, uptake of FIT screening is suboptimal and marked by stark social, regional and ethnic inequalities.^5^ ^6^ Uptake is particularly low in London, where there is an eight-percentage point differential with the national average (ie, 62.1% vs 70.3% (2021/2022)).^5^ This is true despite several interventions having been shown to reduce inequalities and having been implemented as a result. For example, an enhanced reminder letter, sent to non-responders, was previously shown to disproportionately increase uptake among individuals living in more socioeconomically deprived areas and was embedded in the programme as a result.^7^ Two interventions, which are not currently implemented, but are being considered, are text-message reminders and patient navigation (PN).^8 9^

Text message reminders, sometimes referred to as short messaging system reminders, are of particular interest to researchers seeking to reduce inequalities in screening, due to their relatively low cost, non-confrontational nature and the ubiquity of mobile telephones among the adult population (a recent survey by the Office for National Statistics indicated that over 95% of adults living in the UK own a mobile phone).^10^ A systematic review of text message reminders in cancer screening found them to be effective at improving participation rates by up to 15%, with the effectiveness of the reminder being contingent on a number of factors, including the content of the message and the number of messages sent.^11^

PN is another telephone-based intervention, one which involves the provision of personal support, from a trained ‘navigator’, to help patients overcome personal or system-related barriers to healthcare.^12^ As with text message reminders, PN is an intervention that has been of interest to researchers seeking to reduce inequalities in screening, due to the potential to deliver culturally tailored information to individuals whose main language is not English (eg, first-generation migrants). Previous studies have shown that such navigation can increase screening participation.^12^ Indeed, a review and meta-analysis conducted by Nelson *et al* showed a 64% increase in participation in colorectal, breast and cervical screening participation, with a PN intervention.^13^

While strong evidence indicates that both text message reminders and PN are effective, and the aggregate data indicate that PN results in larger effect sizes, few studies have directly compared the effectiveness and cost-effectiveness of these interventions with one another. As such, further research is needed to determine the most effective and cost-effective approach to promoting bowel cancer screening uptake and reducing inequalities in screening. The aim of this study, therefore, is to directly compare these two interventions so as to identify the most effective and cost-effective means to promote uptake of FIT screening in London.

## Methods and analysis

### Study design

This study will be an RCT with three parallel groups. Adults aged 54–74 years, who are registered with a participating general practice, and have not taken part in bowel cancer screening, within 13 weeks of receiving their invitation, will be randomised in equal proportions (1:1:1), to receive either:

1. No intervention.
2. A text-message reminder, followed by additional text-message reminders at 15, 17 and 19 weeks (if there is no response).
3. A text-message reminder, followed by PN calls at 15, 17 and 19 weeks (again, if there is no response).

See online supplemental file 1, figure 1 for an overview of the study design/Consolidated Standards of Reporting Trials diagram.

**Figure 1:**
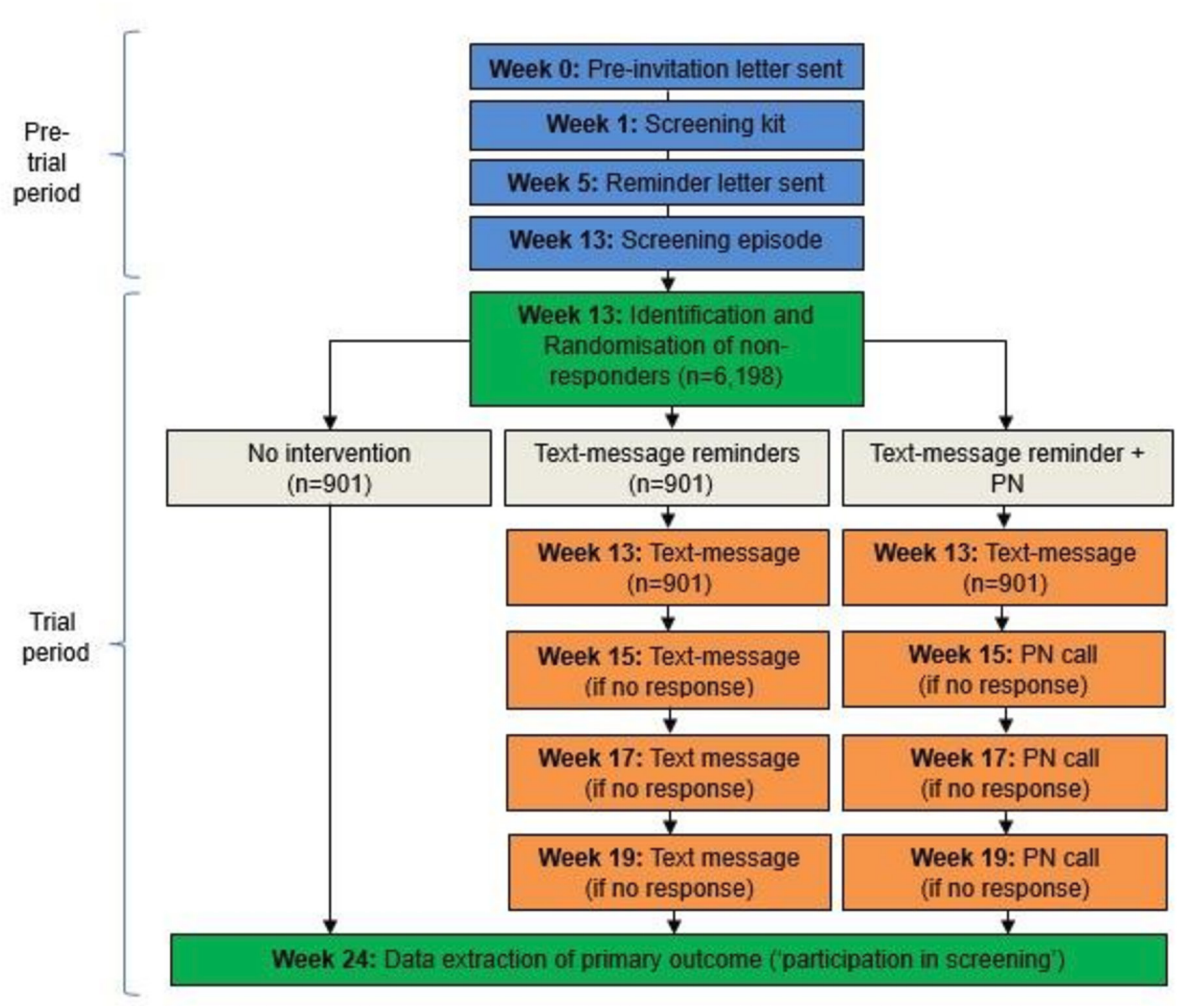
CONSORT diagram detailing the study design. Blue=standard care processes; Green=project-specific processes carried out by London Bowel Cancer Screening Hub; Orange=project-specific processes carried out by iPlato. CONSORT, Consolidated Standards of Reporting Trials; PN, patient navigation.

### Participants and eligibility criteria

Participants will be men and women, aged 54–74 years, who have not returned a bowel cancer FIT screening kit, within 13 weeks of delivery and are registered with a participating general practice located within the London Boroughs of Brent, Ealing, Lambeth, Lewisham, Redbridge and Barking and Dagenham (preselected based on their low screening uptake and CRC survival rates—see table 1). We are deliberately targeting individuals at 13 weeks, as their screening episode is technically closed at this point, and individuals are considered ‘responders’ or ‘non-responders’, depending on whether they have completed the test kit. This means that we are able to focus interventions on non-responders (ie, those who are most likely to benefit from them) and reduce costs by avoiding delivering the interventions to those who would take part without intervention.

**Table 1:** Study setting characteristics: iPlato sign-up, FIT screening uptake and age-standardised mortality rates in London

CCG*	iPlato Sign-up* % (n/n)	FOBt/FIT screening uptake 2019† % (n/n)	FOBt/FIT screening uptake 2019† (compared with London)	Mortality rate† (per 100 000 persons)	Mortality rate† (compared with London)
Brent(Northwest)	94%(49/52)	47.04%(17 364/36 921)	Worse	10.1	Similar
Ealing(West)	88%(66/75)	50.54%(20 782/41 116)	Worse	9.5	Similar
Redbridge(East)	83% (35/42)	50.07%(16 743/33 436)	Worse	8.5	Similar
Barking and Dagenham(East)	97%(33/34)	44.90%(8032/18 491)	Worse	14.8	Worse
Lewisham(South)	94%(33/35)	48.84%(13 524/27 695)	Worse	14.7	Worse
Lambeth(South)	95%(39/41)	45.98%(13 327/28 984)	Worse	11.3	Similar
Total	91.40%(255/279)	48.98%(89 772/1 86 643)			

* Data from iPlato.

† Data from Public Health England—Public Health Profiles.

CCGClinical Commissioning GroupFITfaecal immunochemical test

Exclusions will be made according to ‘type-2 objector’ status, which is defined as: ‘a request, expressed by a registered patient, logged with a general practitioner (GP) practice, to indicate that personal identifiable information relating to them should not be disseminated or published by NHS Digital’.^14^

### Participant recruitment

Eligible adults will be identified on a weekly basis, until the sample size requirement is achieved (approximately 4–8 weeks), by the London Bowel Cancer Screening Hub, which will use data stored on the bowel cancer screening system to identify individuals based on their bowel cancer screening status (ie, whether they are a non-responder) and bowel cancer screening invitation date (ie, to confirm when they were invited for screening, and whether they are a ‘new’ non-responder).

### Consent

To ensure the ecological validity of the study, consent will not be obtained from individual participants. Instead, GPs will provide a proxy consent for iPlato (the text message and PN provider—see below) to contact them on their behalf, using the telephone numbers of patients stored on their clinical system. Potentially eligible participants will be notified that a study is taking place, through patient notification posters, which will be displayed at participating general practices. These posters will be displayed prior to, and throughout, the study, and will inform individuals they can withdraw by notifying reception. At the end of the study, participants will be sent a letter, by the London bowel cancer screening hub, debriefing them that they were involved in a study and the reasons why (see online supplemental appendix 1).

### GP recruitment

GPs will be recruited to the study via email. They will receive an information sheet and consent form from the Joint Screening and Immunisation Lead for Cancer Screening in London (Dr Josephine Ruwende, NHS England), which they will be required to sign and return to the chief investigator (Dr Robert Kerrison, UCL and University of Surrey), in order to participate in the study. Based on previous studies testing similar interventions and recruitment strategies,^15 16^ we expect to recruit ~50% of practices within the London Boroughs of Brent, Ealing, Lambeth, Lewisham, Redbridge and Barking and Dagenham.

### Interventions

As described in Study design section, individuals will be randomised to receive either:

1. no intervention (‘usual care only’).
2. A text-message reminder, 13 weeks after the invitation, with additional text-message reminders at 15, 17 and 19 weeks if there is no response (Individuals in the patient navigation group will receive a text message reminder at 13 weeks, in addition to patient navigation calls at 15, 17 and 19 weeks. This will allow iPlato (the company delivering the patient navigation telephone calls) to confirm which individuals’ telephone numbers are active, and call only those individuals who have an active telephone number. This will reduce the cost of the intervention, by ensuring only those with an active number are contacted (ie, less time spent attempting to contact individuals whose numbers are no longer valid/UpToDate)). (‘text message reminder(s)’).
3. A text-message reminder, 13 weeks after the invitation, followed by PN calls at 15, 17 and 19 weeks if there is no response (‘text message reminder+PN telephone call(s)’).

#### Text message reminders

**Figure 2:**
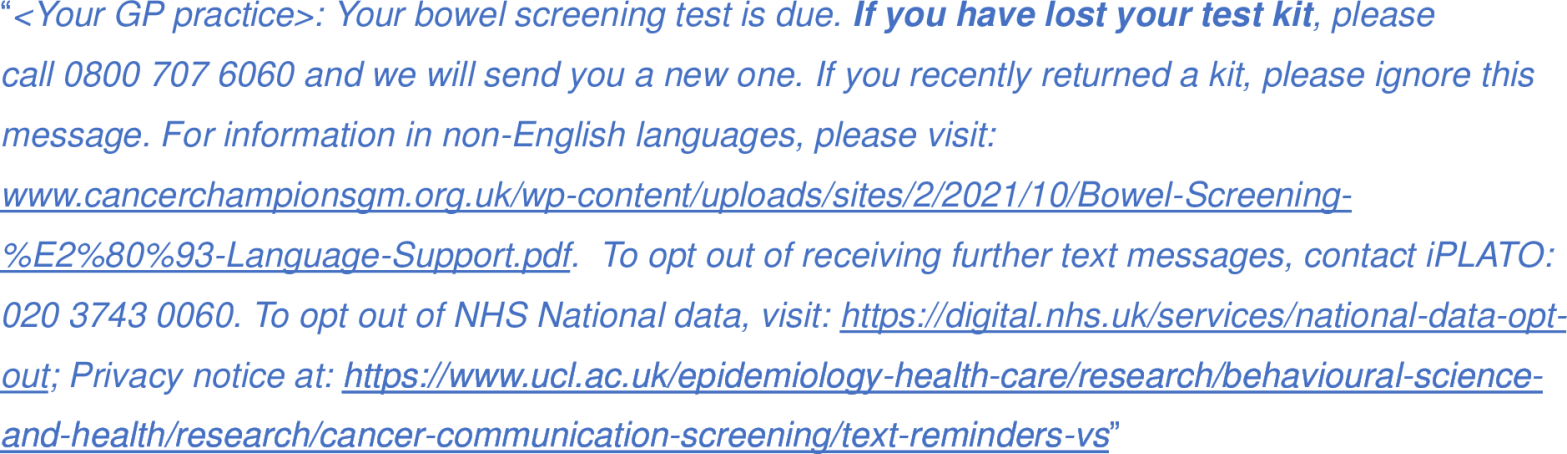
Text message reminder script.

The text message reminders (SF 2, figure 2) for both intervention groups will remind recipients that they have not yet completed their kit, and that they are able to request a new one (should they wish to take part), via Freephone, from the BCSP. The following link, which provides relevant information in different patient languages, will be included in the text message reminder, to support individuals whose first language is not English: www.cancerchampionsgm.org.uk/wp-content/uploads/sites/2/2021/10/Bowel-Screening-%E2%80%93-Language-Support.pdf..

Instructions on how to opt-out of further contact for the study, and wider research, will also be provided.

The decision to include the option for recipients to request a new test kit was based on previous research exploring reasons for not completing an FIT screening kit, which documents one of the reasons as having ‘misplaced’ or ‘lost’ the kit.^17^

#### PN telephone calls

The PN telephone calls will similarly remind recipients that they have not yet completed their kit and will provide them with the opportunity to request a new kit (should they wish to take part) via the London Bowel Cancer Screening Hub. In addition, the PN telephone calls will elicit barriers to participation, which the navigator will provide advice on, to support the individual in completing the test (see online supplemental appendix 2 for a copy of the PN script that will be used for this study). A record of all calls, and the barriers discussed, will be documented, to elucidate which barriers are successfully addressed by the PN calls (this will be confirmed by request and completion of a test kit).

#### Intervention development

The wording for the text message reminders was decided through consultation between the director of the London Bowel Cancer Screening Hub (NKD), the Commissioner for Cancer Screening Services in London (JR), patients and members of the public and the academic research team (TD, RK, SD and CvW).

The PN script was developed by the UCL research team, using Centers for Disease Control and Prevention (CDC) guidelines.^18^ As per the CDCs recommendations, the script begins with the navigator clarifying the nature of the invitee’s concern, to ensure that the PN process is addressing their true needs. Thereafter, the script instructs the navigator to empathise with the patient and acknowledge that their concerns are valid. In the final stage, the script instructs the navigator to address the concerns with transparency and specificity.

To ensure navigators have sufficient knowledge and understanding to deliver navigation for bowel cancer screening, they will be required to complete Health Education England training on Population Screening and shared decision-making. As such, they will be aware of the benefits and drawbacks of screening and will be able to discuss these with participants. The decision to participate will be an informed one and should the patient express that they do not want to take part, this decision will be respected by the navigator. Once all patient concerns have been addressed, the script instructs the navigator to assist the invitee in a plan to participate (eg, by ordering a new FIT kit, if the original kit has been mislaid).^19^

### Intervention delivery

Both interventions will be delivered by iPlato: an mHealth company who have previous experience delivering text message reminders and PN for other cancer screening programmes (eg, breast) and are used for general services by over 90% of London’s general practices.^20^ The callers are experienced navigators who have previously delivered PN to breast cancer screening non-attenders. They regularly attend training, provided by Health Education England, and will have completed the following Health Education England programmes, before the study commences: (1) behaviour change literacy for individuals and workforce; (2) cancer in the community; (3) communicating with empathy (making the most of listening); (4) data security awareness; (5) population screening; (6) shared decision-making; (7) the changing story of cancer and (8) cultural competence. Calls will not be recorded.

To protect patient confidentiality, navigators will not leave messages (as these messages may be intercepted by others) or reveal the reason for their call to others. A dedicated telephone number will be set up for the study and will not be withheld from participants.

The official start date for the research was August 2021. However, due to disruptions to bowel cancer screening delivery (ie, temporary suspension of the programme, following the onset of the COVID-19 pandemic), the study was paused while the programme went through recovery (ie, addressed backlogs in screening invitations and follow-up colonoscopy of abnormal results). The study recommenced in September 2022, with a focus on obtaining the necessary ethical approvals for the research (eg, sponsor approval, NHS ethics). Having obtained the necessary approvals, the planned start date for recruitment is set for July 2024. The trial will run for approximately 8 weeks (1 July 2024–25 August 2024), with a follow-up period of 11 weeks (25 August 2024–9 September 2024) for kits to be returned, following the delivery of the final intervention.

In line with standard practice, iPlato will use a numerical score structure to assess participant satisfaction with the calls (see online supplemental appendix 3). An open question will also be administered, via text message, to obtain more detailed feedback/free-text responses. Responses will be reported using descriptive statistics to describe the means and distributions of the numerical structures. Free-text responses will be used to supplement the scores, to explore reasons for high and low satisfaction. The score structure will be administered via text message immediately after the call.

### Blinding and randomisation

This will be a single-blind RCT. The study investigators will not know which individuals have been assigned to the intervention or control groups, until the end of the trial, when all data have been collected and anonymised. Individuals registered at a participating GP practice will not be informed that they are participants in a study, during the study period; however, those receiving an intervention cannot be blinded to the condition they have been allocated, due to the overt nature of the interventions (hence, we describe this as a ‘single-blind’ RCT). Randomisation of individuals to study groups will be handled by iPlato, using simple, computerised, pseudorandom allocation methods. Individuals will be randomised in a 1:1:1 ratio to either the intervention or control condition, after 13 weeks of their screening episode, irrespective of whether or not they have a mobile number registered on their GP’s clinical system.

### Outcomes

The primary outcome will be participation in screening (defined as the return of a completed FIT kit within 24 weeks of dispatch (episodes ‘reopened’ between 13 and 24 weeks contribute towards uptake)) and will be compared between the three trial arms. The secondary outcome will be the kit result (defined as ‘normal’ or ‘abnormal’) and will also be compared between the three trial arms. This will allow us to determine whether the interventions have not only increased participation but increased participation of those with pathology, specifically.

### Data collection

At the end of the study (‘week 24’), the London Bowel Cancer Screening Hub will extract the following data on study participants, from the available bowel cancer screening system: sex, age, index of multiple deprivation quintile (derived from the participant’s postcode), adequate completion (ie, ‘kit returned’ (yes/no)) and bowel cancer screening result (normal/abnormal). The data will then be anonymised and analysed by a data analyst at the London Hub.

### Sample size calculation

For the control, text-message reminder and PN groups, we anticipate that 1.0%, 3.2% and 15.0% of participants (respectively) will complete a kit by week 24 (this is based on data provided by NHS England, as well as previous research conducted with the London BCSP) (Personal Communication). We subsequently designed our sample size calculation to provide sufficient power to allow us to test for the smallest possible difference, between any two groups, of 2.2 percentage points (ie, 1% in the control vs 3.2% in the text message reminder group). As three separate comparisons are planned for the main analysis (control vs text message reminders; control vs PN; text message reminders vs PN), we used an adjusted significance level (ie, 0.015) for our sample size calculation (as per the Bonferroni correction method, which takes into account inflation of the alpha statistic, caused by multiple comparisons), so as to maintain an overall 5% probability for any false positive result. To achieve 80% power at this level, our study requires 901 subjects per group (2703 recruits in total; see table 2 for details).

**Table 2:** Sample size calculations

	P1	P2	Alpha*	Beta	n (per arm)	n (total)
Comparison 1						
No intervention† versusText-message reminders‡	0.0098	0.0318	0.015	0.2	901	2703
Comparison 2						
No intervention† versusText-message reminder+PN§	0.0098	0.1334	0.015	0.2	92	276
Comparison 3						
Text-message reminders versusText-message reminder+PN§	0.0318	0.1334	0.015	0.2	156	468

* An adjusted Alpha of 0.015 has been used to account for inflation of the statistic, resulting from multiple comparisons (ie, 0.05 / 3).

† Expected uptake based on 2019 data for FOBt/FIT, 13-24 weeks after invitation (PHE, personal communication).

‡ Expected uptake for text reminders based on the results of the service evaluation for the Southeast London breast screening service (iPlato, personal communication).

§ Expected uptake for text reminders and patient navigation based on the results of the service evaluation for the Southeast London breast screening service (iPlato, personal communication).

FITfaecal immunochemical test

### Data analysis

Descriptive statistics (ie, frequencies and percentages) will be used to report the demographic characteristics, primary outcome and secondary outcome by study group. Differences between study groups, with respect to the primary outcome, will be assessed using univariate and multivariate logistic regression, before and after adjusting for demographic covariates. As there are three study groups, pairwise comparisons will be performed to test for differences between any two groups. These analyses will be performed on an intention-to treat basis and per-protocol basis so that both the ‘real-world’ impact and the ‘potential’ impact of the interventions can be assessed (not all individuals will have a valid mobile number recorded on the GP clinical system and so not all intervention participants will be able to receive the text message reminder and PN interventions). To conduct the per-protocol analyses, we will include individuals with a mobile telephone number stored on the clinical system for the control group, individuals who successfully received a text message reminder for the text message reminder group, and individuals who answered the phone in the PN group. We will also perform subgroup analyses, to explore the effectiveness of the interventions, by demographic characteristics, on an intention-to-treat and per-protocol basis. Finally, we will perform a cost-effectiveness analysis, to estimate the costs of the interventions per additional person screened. This will involve dividing the total cost of intervention, by the number of additional people screened in each intervention arm, over and above the number screened in the control arm.

All analyses will be performed by using SSPS statistics (V.27.0).

### Patient and public involvement

#### Design of the research

It is important that the interventions are delivered in a way that is acceptable to members of the public. For this reason, we consulted members of the public for their advice regarding the wording and timing of the text-message reminders and PN telephone calls. We conducted a series of patient and public involvement discussions in order to develop the text message and PN content. This involved consultation with service users to determine the key information for inclusion and the most effective language for delivery. To ensure cultural sensitivity, the PN training included completion of core cultural competence other relevant training modules, available via the NHS e-learning platform. We also ensured iPlato would provide multilingual staff for the delivery of PN.

#### Management of the research

No involvement.

#### Analysis of results and dissemination of findings

It is important that the research outputs are accessible to members of the public. For this reason, we will consult members of the public for their advice regarding the wording of the manuscripts, prior to submission for publication.

### Debrief plan

At the end of the study, participants will be sent a letter debriefing them about the study (see online supplemental appendix 1). Participants will be debriefed by the London bowel screening hub, who have access to the first name, last name, NHS number and home address of participants (neither iPLATO, nor the researchers, will have access to participants’ home address, during the study). The letters and envelopes will be printed and distributed in-house, by the London bowel cancer screening hub, so that details are not shared externally. In the debriefing letter, we include an invitation to participate in a short follow-up interview for those who received a text message or telephone call reminder, to allow process evaluation of the interventions (see online supplemental appendix 4 for interview schedule).

## Ethics and dissemination

### Ethics

Approvals to conduct the research have been obtained from University College London’s Joint Research Office (Ref: 150666), the Screening Research, Innovation and Development Advisory Committee (‘RIDAC’, Ref: 2223 014 BCSP Kerrison), the Health Research Authority (Ref: 22/WM/0212) and the Confidentiality Advisory Group (Ref: 22/CAG/0140).

### Trial registration

This trial is registered with the International Standardised Randomised Controlled Trials Number Registry (ISRCTN): https://www.isrctn.com/ISRCTN17245519..

### Dissemination plan

The results of this RCT will be reported in a peer-reviewed journal and presented at scientific conferences. In addition, the funding body and the stakeholders (in particular the management of the BCSP) will be informed of the results prior to publication, in case the results merit action in terms of policy or practice.

This study will provide evidence on the effectiveness and cost-effectiveness of text message reminders and PN to promote the uptake of FIT in low-uptake areas of London. The findings will be of considerable interest to the BCSP, which is attempting to reduce inequalities in bowel cancer screening participation, between London and the rest of the country. The results are likely to inform whether the programme uses telephone PN and/or text message reminders for bowel cancer screening in the future.

In addition to informing the future use of these interventions within the BCSP, results from the present trial might offer a short-term solution to mitigating the COVID-19 pandemic on bowel cancer survival (it is well established that patients have not been seeking help for bowel cancer-related symptoms during lockdown, and that fewer bowel cancer cases were diagnosed in 2020). Studies to improve early cancer detection and increase screening rates are necessary in terms of potentially reducing these risks.

## supplementary material

10.1136/bmjopen-2023-079482 online supplemental file 1
