# Supplementary material for: Comparing the effectiveness and cost-effectiveness of text-message reminders and telephone patient navigation to improve the uptake of faecal immunochemical test screening among non-responders in London: a randomised controlled trial protocol
Source: BMJ Open. 2024 Jun 22;14(6):e079482. doi: 10.1136/bmjopen-2023-079482 (PMC11328611; doi:10.1136/bmjopen-2023-079482)
Supplement: online supplemental file 1 [file bmjopen-14-6-s001.pdf]

Appendix 1. Debrief letter

<DD> <Month> <Year>

Dear <Title> <First Name> <Last Name>,

You were recently sent a letter to take part in the NHS Bowel Cancer Screening Programme. At the time you received your letter, a study was taking place to see whether text message and telephone reminders can increase the number of people who return a bowel cancer screening kit. The study was led by UCL, funded by NHS England, and reviewed by an NHS Research Ethics Committee.

You were included in the study, and so may have received one or more text or telephone reminders as part of your involvement. You were not directly informed that the study was taking place, as telling people they are part of a study can lead to them returning their bowel screening kit by itself. Your GP was aware of the study, and put up posters to say it was taking place. Special approvals were granted to include you in the study without your knowledge.

We are writing to you now, as we have a duty to notify participants who were unknowingly included in research. A more detailed explanation of the research follows:

You were part of either a “follow-up contact group” or a “control group”. If you were allocated to a follow-up contact group, you will have either received one or more text message and / or telephone reminders. If you were in the control group, you will have received no text or telephone reminders. We expected that participants in the follow-up contact group would be more likely to participate in bowel cancer screening, compared to the control group. This is because participants in the follow-up contact group were given additional support, such as the option to request a new test kit if they needed it, or a conversation with a navigator to discuss any issues they might encounter with screening.

The study found that text and telephone reminders did / did not improve participation. On this basis, it has been recommended that they do / do not become routine practice. This will allow the NHS to use it’s resources more efficiently.

If you received a text message or telephone call reminding you to complete your test kit, we would like to speak with you about your experience of this. Please contact Dr Rob Kerrison on r.kerrison.13@ucl.ac.uk to participate.

Thank you for your participation. If there is anything you would like to discuss in relation to this study, please feel free to do so by contacting the researcher at: Robert.kerrison.13@ucl.ac.uk

To opt out of having your confidential patient information being used for future research and planning, please visit: https://digital.nhs.uk/services/national-data-opt-out

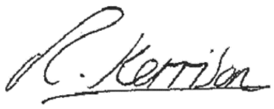

Dr Robert Kerrison, Senior Research Fellow, University College London

Appendix 2

Patient navigation script

Introduce yourself

Step 1. Hello. My name is <NAME> and I'm calling from iPlato.

STEP 2. Establish whether they are available to talk:

I'm calling about your bowel cancer screening kit [should have received kit 13 weeks earlier]. Is now a convenient time to speak?

If the patient states: "Yes"

1) Move on to Step 3 (explain why you're calling)

If the patient states: "No"

1) **Reschedule:** "That's okay. Is there a time, later in the week, when I can call you instead?"

Thank them (if the patient does not want to reschedule): "That's okay. If you have any concerns at all, please call the freephone bowel cancer screening helpline on: **020 3743 0060**"

Step 3. Our records show you've received a bowel cancer screening test kit in the post and have not returned it. Is this true?"

Patient states, "Yes, I did receive a test kit in the mail but I didn't think I needed to be screened"

Ask clarifying questions to better understand the patient's concern

- I'd be happy to help you with this. But first, can you help me understand why you don't think you need to be screened?"

Patient states, "I don't feel sick and I don't have any family history of bowel cancer."

Empathize with the patient to convey that you understand their concern

- I hear you. You're not alone. Many people think they don't need screening. Even if you don't feel sick, screening is important."

Respond directly to address the patient's concerns

- At age 60, both men and women should start getting screened for colon cancer, regardless of family history."
- Many people with bowel cancer have no family history of it. You can have colon cancer and not feel sick. By screening we can detect it at an early stage."
- With regular, recommended screening, the mortality of colon cancer would be reduced dramatically."

Other concerns and responses.

Patient states "I have received a test kit but I'm nervous about it saying I have cancer"

Respond with

- I understand your concern and you're not alone. Being nervous about a positive result is not unusual"
- However if diagnosed early the condition will be easier to treat"
- It is unlikely you will have it but early diagnosis is going to make it more treatable"
- 1 in 15 males in the UK will be diagnosed with cancer (6.7%). (20)

- Bowel cancer survival is improving and has more than doubled in the last 40 years in the UK. (20)
- Medicines and food do not interfere with the test. As such it tends to be more accurate and have fewer false positive results than other tests so it’s unlikely to give you a false positive or false negative. (21)

Patient states “I’m embarrassed about this”

Respond with

- I understand lots of people feel embarrassed about collecting a sample

Patient states “No, I haven’t received my kit”

Request a new kit for the patient.

Patient states “yes, I received my kit and returned it”

Respond with

- When did you send out your kit?
  If less than 2 weeks ago assume it will come through the post soon.
  If more than 2 weeks ago assume something happened and request a new kit.

Questions

Q. How the Test is Performed

A. You will be given the test to use at home. Be sure to follow the instructions provided. Most tests have the following steps:

1. The easiest way to collect the sample is to use an old plastic container, line it with toilet paper and put it in the toilet. And then have a poo into the container. Do not wee into the container. And do not let your poo touch the water or the toilet. Once you have collected your poo:
2. dip the stick from the testing kit in the poo
3. make sure the end is covered with poo
4. put the stick back in the tube and twist it shut
5. write the date on the side of the tube
6. put the tube into the sample bag. (21)

Q. How does the test know if I’ve got cancer?

A. FIT detects hidden blood from the lower intestines which can be an early sign of cancer. (21)

Q. When would I get my result?

A. You should expect your results within 1 or 2 weeks. (21)

Appendix 3. Patient satisfaction survey

Text. Thank you for speaking with us today. We would like to ask you three quick questions regarding your experience. Please click the following link to take part <Link>:

Question 1. On a scale of 1 - 5, where 1 is 'not at all comfortable' and 5 is 'very comfortable', how comfortable did you feel about being called by our team today?

Question 2. Thank you. On a scale of 1 - 5, where 1 is 'not at all satisfied' and 5 is 'very satisfied', how satisfied were you with the way the conversation progressed?

Question 3. Thank you. If have any feedback, please respond to this text message with any comments you may have.

Appendix 4 - Interview schedule for process evaluation

AIM

Short interview to understand why the interventions were effective.

TEXT MESSAGE REMINDERS

For those people who didn’t initially return their kit, but did following a text message reminder.

- What was it about receiving a text message that led you to return your kit?
- Did you request a new kit?
- Did you return the kit that you already had?
- Did you click on any links contained within the text message?
- Did you call the freephone number for advice?
- If so; what was your experience of this?

PATIENT NAVIGATION

For those people who returned their kit following a call from iPlato.

- Can you tell me how iPlato helped you to complete the test?
- Did iPlato request a new kit for you?
- Did iPlato give you any advice on completing the kit?
