# Supplementary material for: Comparing the effectiveness and cost-effectiveness of text-message reminders and telephone patient navigation to improve the uptake of faecal immunochemical test screening among non-responders in London: a randomised controlled trial protocol
Source: BMJ Open. 2024 Jun 22;14(6):e079482. doi: 10.1136/bmjopen-2023-079482 (PMC11328611; doi:10.1136/bmjopen-2023-079482)
Supplement: Supplementary file 2 [file bmjopen-2023-079482.reviewer_comments.pdf]

PEER REVIEW HISTORY

BMJ Open publishes all reviews undertaken for accepted manuscripts. Reviewers are asked to complete a checklist review form (**http://bmjopen.bmj.com/site/about/resources/checklist.pdf**) and are provided with free text boxes to elaborate on their assessment. These free text comments are reproduced below.

ARTICLE DETAILS

**TITLE (PROVISIONAL)**	Comparing the effectiveness and cost-effectiveness of text-message reminders and telephone patient navigation to improve the uptake of faecal immunochemical test screening among non-responders in London: A randomised controlled trial protocol.
**AUTHORS**	Duffy, Thomas; Gil, Natalie; Siddique, Benzeer; Duffy, Stephen; Prentice, Andrew; Marshall, Sarah; Djedovic, N; Lewis, Michael; Ruwende, Josephine; von Wagner, Christian; Kerrison, Robert

VERSION 1 – REVIEW

**REVIEWER**	Belinda Goodwin<br>University of Southern Queensland
**REVIEW RETURNED**	10-Nov-2023

**GENERAL COMMENTS**	The manuscript provides a very clear and thorough description of the protocol for a RCT testing the effectiveness of SMS and patient navigation phone calls in increasing in bowel cancer screening in areas of London marked by lower-than-average participation rates.<br><br>I just have a few minor comments for the authors consideration:<br><br>A very brief description of the English program, including age and GP registration eligibility will provide context for the sample described in the methods especially for the international reader. It would also help to describe the way in which GP and Hub data are integrated/linked in the UK. Readers from settings where general practice does not play a key role in the delivery of screening programs may wonder how the patient data (e.g., mobile phone numbers) from consenting practices are shared with the London Bowel Cancer Screening Hub.<br><br>I'm curious as to why the SMS refers recipients to order a new kit. Is this based on the fact the kit may have expired or been lost in the meantime? The message almost implies that to participate, the person needs to order a new kit. If this is not the case, it could lead to some wastage. Not a flaw in the study design by any means but it might be worth explaining the reasoning behind including this in the message.<br><br>Line 58, page 6: "we will include individuals with mobile telephone…" missing an "a".<br><br>It wasn't entirely clear how the sensitivity analysis would estimate the costs of interventions. As I understand it, a sensitivity analysis examines the effects of independent variables on the dependent variable (in this case, kit return) under different assumptions.

	Estimating the cost or cost-benefit ratio of each intervention, I would have thought would be a separate analysis. Can I suggest providing just a little more detail on the variables for this analysis and how they will be modelled.<br><br>Page 9, 4.1: Could the author provide just a little more detail on how members of the public consulted and how many?<br><br>The proposed timing of the trial is well described. However, I note that the journal requests that dates of the trial are specified – could the authors add proposed start and finish dates?

**REVIEWER**	Ivo Vlaev<br>Warwick Business School
**REVIEW RETURNED**	05-Dec-2023

**GENERAL COMMENTS**	The provided academic paper outlines a randomised controlled trial (RCT) to compare the effectiveness of text-message reminders and patient navigation (PN) in increasing the uptake of faecal immunochemical test (FIT) screening for colorectal cancer in London. Despite existing interventions, uptake is low, particularly in London, with disparities across social, regional, and ethnic groups. The study will target individuals who have not responded to screening invitations within 13 weeks. It will explore whether text messages or personalised telephone support can effectively increase participation, especially among communities with historically low screening rates. This research is significant for public health as it aims to increase early detection of colorectal cancer and improve survival rates.<br><br>The design of the study incorporates behavioural science principles effectively, especially the use of reminders and personalised navigation, which are grounded in the understanding of human behaviour and the barriers to action. The intervention targets known points of friction such as forgetfulness and lack of understanding, which can prevent individuals from participating in health screenings.<br><br>The use of text-message reminders leverages the widespread ownership of mobile phones and the habitual nature of checking messages, thereby using an existing behaviour pattern to nudge participants towards action. Patient navigation addresses more complex barriers such as language, fear, or misunderstanding, offering tailored support. This dual approach is likely to address a broad spectrum of reasons for non-participation.<br><br>The study design is methodologically sound with a clear RCT structure, targeting a specific population that has shown low uptake rates. The consideration of ethical aspects, data handling, and detailed planning for interventions and outcomes indicates thorough planning.<br><br>The paper could benefit from a bit more detailing how the content of the text messages and the training of patient navigators are developed to ensure cultural sensitivity and effectiveness.<br><br>Additionally, more information on why process evaluation could not be embedded in the in the design and compared would be beneficial. For example, why a number of individuals are not contacted to explore (in a structured way) how the intervention affected their minds and actions (which may shed light on how

	different components of the intervention affected the outcomes)?<br><br>Finally, the study could also consider potential biases introduced by not obtaining individual consent and relying on proxy consent from GPs, which may affect the ecological validity of the study.

VERSION 1 – AUTHOR RESPONSE

Reviewer: 1

RC. The manuscript provides a very clear and thorough description of the protocol for a RCT testing the effectiveness of SMS and patient navigation phone calls in increasing in bowel cancer screening in areas of London marked by lower-than-average participation rates.

I just have a few minor comments for the authors consideration:

AR. We would like to thank Reviewer 1 for taking the time to read our manuscript and for their kind words. Please find our responses to their comments below.

RC. A very brief description of the English program, including age and GP registration eligibility will provide context for the sample described in the methods especially for the international reader. It would also help to describe the way in which GP and Hub data are integrated/linked in the UK. Readers from settings where general practice does not play a key role in the delivery of screening programs may wonder how the patient data (e.g., mobile phone numbers) from consenting practices are shared with the London Bowel Cancer Screening Hub.

AR. Thank you for your comments and for your suggestion to include a description of the English bowel screening programme and how General Practice facilitates this. We have added a paragraph describing the operational aspects of the programme, in more detail:

Page 3. "In England, the National Health Service (NHS) began offering biennial FIT screening to adults aged 60-74 years in 2019. The screening programme, referred to as the NHS Bowel Cancer Screening Programme (BCSP), is delivered through five national hubs, who send kits to patients registered with a general practice within their region. The delivery of invitations is an automated and centralised process in which information held by the patient's general practice, including their date of birth, name and address, are shared with the BCSP, to facilitate the process. Date of birth is used to identify individuals who are eligible for screening, while name and address are used to send the invitations to those individuals home address. An age extension to the programme is underway, with the aim of all adults, aged 50-59, receiving an invitation by 2025. At the time of writing (January 2024), the programme has been extended to include adults aged 54-59 years."

RC. I'm curious as to why the SMS refers recipients to order a new kit. Is this based on the fact the kit may have expired or been lost in the meantime? The message almost implies that to participate, the person needs to order a new kit. If this is not the case, it could lead to some wastage. Not a flaw in the study design by any means but it might be worth explaining the reasoning behind including this in the message.

AR. Thank you for highlighting the need to clarify the information conveyed within the SMS regarding ordering a new test kit. The intervention is being delivered 13 weeks after the initial invitation and test kit and, as such, we believe it to be likely that many people will have lost their original test kit. This is based on evidence that suggests "having misplaced or loss of the kit" is one of the reasons for non-participation. By offering the opportunity for a replacement kit, we hope to overcome this particular barrier.

We have added a sentence explaining this, to our manuscript, and amended the wording of the text message for clarity:

Page 6. “The decision to include the option for recipients to request a new test kit was based on previous research exploring reasons for not completing a FIT screening kit, and documented one of the reasons as having ‘misplaced’ or ‘lost’ the kit (16)”

Page 14. “: Your bowel screening test is due. If you have lost your test kit, please call 0800 707 6060 and we will send you a new one. If you recently returned a kit, please ignore this message. For information in non-English languages, please visit: www.cancerchampionsgm.org.uk/wp-content/uploads/sites/2/2021/10/Bowel-Screening-%E2%80%93-Language-Support.pdf. To opt out of receiving further text messages, please contact iPLATO: 020 3743 0060. To opt out of NHS National data, visit: https://digital.nhs.uk/services/national-data-opt-out; Privacy notice at: https://www.ucl.ac.uk/epidemiology-health-care/research/behavioural-science-and-health/research/cancer-communication-screening/text-reminders-vs”

RC. Line 58, page 6: “we will include individuals with mobile telephone…” missing an “a”.

AR. Thank you for highlighting this. We have corrected as advised.

RC. It wasn’t entirely clear how the sensitivity analysis would estimate the costs of interventions. As I understand it, a sensitivity analysis examines the effects of independent variables on the dependent variable (in this case, kit return) under different assumptions. Estimating the cost or cost-benefit ratio of each intervention, I would have thought would be a separate analysis. Can I suggest providing just a little more detail on the variables for this analysis and how they will be modelled.

AR. Thank you for suggesting the need for additional detail and clarity regarding the sensitivity analysis and variables for analysis and modelling. We have now updated the paper to refer to this as a cost-effectiveness analysis. An outline of this is documented below.
The analysis is designed to determine the cost of the interventions, per additional person screened. Cost per additional person screened will be calculated by dividing the total cost of intervention, by the number of additional people screened in each intervention arm, compared with the control arm.
We have updated our manuscript to reflect this:
Page 9. “Finally, we will perform a cost-effectiveness analysis, to estimate the costs of the interventions per additional person screened. This will involve dividing the total cost of intervention, by the number of additional people screened in each intervention arm, over and above the number screened in the control arm.”

RC. Page 9, 4.1: Could the author provide just a little more detail on how members of the public consulted and how many?

AR. Thank you for highlighting the need for additional detail regarding the patient and public (PPI) involvement of the study. We have now amended the PPI section as follows:

Page 9. “It is important that the interventions are delivered in a way that is acceptable to members of the public. For this reason, we consulted members of the public for their advice regarding the wording and timing of the text-message reminders and PN telephone calls. We conducted a series of patient and public involvement (PPI) discussions in order to develop the Text Message and Patient Navigation content. This involved consultation with service users to determine the key information for inclusion and most effective language for delivery. To ensure cultural sensitivity, the Patient Navigation training included completion of Core Cultural Competence other relevant training modules, available via the NHS e-learning platform. We also ensured iPlato would provide multilingual staff for the delivery of Patient Navigation.”

RC. The proposed timing of the trial is well described. However, I note that the journal requests that dates of the trial are specified – could the authors add proposed start and finish dates?

AR. Thank you for your question. The dates of the trial are 01/04/2024 – 09/08/2024. We have updated the manuscript to explain this:

“The trial will run for approximately eight weeks (01/04/2024 – 25/05/2024), with a follow-up period of 11 weeks (25/05/2024 – 09/08/2024) for kits to be returned, following the delivery of the final intervention.”

Reviewer: 2
RC. The provided academic paper outlines a randomised controlled trial (RCT) to compare the effectiveness of text-message reminders and patient navigation (PN) in increasing the uptake of faecal immunochemical test (FIT) screening for colorectal cancer in London. Despite existing interventions, uptake is low, particularly in London, with disparities across social, regional, and ethnic groups. The study will target individuals who have not responded to screening invitations within 13 weeks. It will explore whether text messages or personalised telephone support can effectively increase participation, especially among communities with historically low screening rates. This research is significant for public health as it aims to increase early detection of colorectal cancer and improve survival rates.

The design of the study incorporates behavioural science principles effectively, especially the use of reminders and personalised navigation, which are grounded in the understanding of human behaviour and the barriers to action. The intervention targets known points of friction such as forgetfulness and lack of understanding, which can prevent individuals from participating in health screenings.

The use of text-message reminders leverages the widespread ownership of mobile phones and the habitual nature of checking messages, thereby using an existing behaviour pattern to nudge participants towards action. Patient navigation addresses more complex barriers such as language, fear, or misunderstanding, offering tailored support. This dual approach is likely to address a broad spectrum of reasons for non-participation.

The study design is methodologically sound with a clear RCT structure, targeting a specific population that has shown low uptake rates. The consideration of ethical aspects, data handling, and detailed planning for interventions and outcomes indicates thorough planning.

AR. We would like to thank Reviewer 2 for taking the time to read our manuscript and for their kind words about our research. Please find our response to the comments below.

RC. The paper could benefit from a bit more detailing how the content of the text messages and the training of patient navigators are developed to ensure cultural sensitivity and effectiveness.

AR. Thank you for highlighting the need to provide additional detail surrounding the development of interventions and how we ensured optimum cultural sensitivity. Firstly, we conducted a series of patient and public involvement (PPI) discussions in order to develop the Text Message and Patient Navigation content (see Pages 9 and 10, updated in response to reviewer 1’s comments). This involved consultation with service users to determine the key information for inclusion and most effective language for delivery. To ensure cultural sensitivity, the Patient Navigation training included completion of Core Cultural Competence other relevant training modules (see Pages 9 and 10, updated in response to Reviewer 2’s comments), available via the NHS e-learning platform. We also ensured iPlato would provide multilingual staff for the delivery of Patient Navigation:

Pages 9 and 10. “We conducted a series of patient and public involvement (PPI) discussions in order to develop the Text Message and Patient Navigation content. This involved consultation with service users to determine the key information for inclusion and most effective language for delivery. To ensure cultural sensitivity, the Patient Navigation training included completion of Core Cultural Competence other relevant training modules, available via the NHS e-learning platform. We also ensured iPlato would provide multilingual staff for the delivery of Patient Navigation.”

RC. Additionally, more information on why process evaluation could not be embedded in the in the design and compared would be beneficial. For example, why a number of individuals are not contacted to explore (in a structured way) how the intervention affected their minds and actions (which may shed light on how different components of the intervention affected the outcomes)?

AR. Thank you for your suggestion to include additional information regarding process evaluation. We recognise the need for a more structured exploration of how the intervention may have worked to affect actions of participants. We will invite participants to take part in a process evaluation interview, via a debrief letter, which will be sent to participants at the end of the study, and include the following message:

Page 9. “If you received a text message or telephone call reminding you to complete your test kit, we would like to speak with you about your experience of this. Please contact Dr Rob Kerrison on r.kerrison.13@ucl.ac.uk to participate.”

We have developed a draft interview schedule for use when conducting these interviews (See Supplementary Files).

RC. Finally, the study could also consider potential biases introduced by not obtaining individual consent and relying on proxy consent from GPs, which may affect the ecological validity of the study.

AR. Thank you for highlighting the need to consider the potential biases. We have now added a detailed strengths and limitations of this study section to the protocol.

“Strengths & limitations of this study:
• This is a Randomised Controlled Trial using individual level data, as opposed to area-level data, increasing the studies ability to attribute increases in uptake, directly to the intervention.
• The study focusses on ethnically diverse areas, increasing the generalisability of the research to other parts of the United Kingdom.
• The study relies on General Practices to consent to the study, which introduces an element of self-selection bias.
• The text message reminders will be delivered in English, which may limit the equitability of the intervention and undermine its effectiveness.
• Not every participant will have a mobile telephone number, which could undermine results. The study will use intention to treat analysis to compare individuals across groups, who have a mobile number recorded on the GP system.”

VERSION 2 – REVIEW

**REVIEWER**	Belinda Goodwin<br>University of Southern Queensland
**REVIEW RETURNED**	07-Feb-2024

**GENERAL COMMENTS**	Thanks for addressing my comments. I'm excited to see the results of the trial!

**REVIEWER**	Ivo Vlaev<br>Warwick Business School
**REVIEW RETURNED**	01-Mar-2024

**GENERAL COMMENTS**	The authors have fully addressed the comments and suggestions.
